# Whole-Genome Sequence of *Photobacterium damselae* subsp. *piscicida* Strain 91-197, Isolated from Hybrid Striped Bass (*Morone* sp.) in the United States

**DOI:** 10.1128/genomeA.00600-17

**Published:** 2017-07-20

**Authors:** Yuki Teru, Jun-ichi Hikima, Tomoya Kono, Masahiro Sakai, Tomokazu Takano, John P. Hawke, Haruko Takeyama, Takashi Aoki

**Affiliations:** a Department of Biochemistry and Applied Biosciences, Faculty of Agriculture, University of Miyazaki, Gakuen Kibanadai-nishi, Miyazaki, Japan; b National Research Institute of Aquaculture, Japan Fisheries Research and Education Agency, Minami-Ise, Mie, Japan; c Department of Pathobiological Sciences, School of Veterinary Medicine, Louisiana State University, Baton Rouge, Louisiana, USA; d Department of Life Science and Medical Bioscience, Waseda University, Wakamatsu-cho, Shinjuku-ku, Tokyo, Japan; e Integrated Institute for Regulatory Science, Research Organization for Nao and Life Innovation, Waseda University, Waseda Tsurumaki-cho, Shinjuku-ku, Tokyo, Japan

## Abstract

*Photobacterium damselae* subsp. *piscicida* is a causative bacterium of fish pasteurellosis, which has caused serious economic damage to aquaculture farms worldwide. Here, the whole-genome sequence of *P. damselae* subsp. *piscicida* 91-197, isolated in the United States, suggests that this genome consists of two chromosomes and two plasmids.

## GENOME ANNOUNCEMENT

*Photobacterium damselae* subsp. *piscicida* is the causative agent of acute fish pasteurellosis, a very serious bacterial septicemia. This disease, which has caused serious economic damage to aquaculture farms around the world, was first observed in natural populations of white perch (*Morone americanus*) and striped bass (*Morone saxatilis*) in 1963 in the Chesapeake Bay, United States (1). It has since been observed in yellowtail (*Seriola quinqueradiata*) in Japan (2–4) and in gilthead sea bream (*Sparus aurata*) and sea bass (*Dicentrarchus labrax*) in Europe (5, 6). In a previous study, we determined the complete genome sequence of the Japanese isolate OT-51443 (7). To identify potential differences in the genomic sequences between bacterial isolates from the United States and Japan, we characterized the whole-genome sequence of *P. damselae* subsp. *piscicida* strain 91-197 from the United States.

*P. damselae* subsp. *piscicida* strain 91-197 was isolated from a hybrid striped bass (*Morone* sp.) in the United States (8). Strain 91-197 was cultured in heart infusion broth at 25°C for 20 h. Bacterial cells were collected by centrifugation at 8,000 × *g* for 10 min. Genomic DNA was extracted from the bacterial pellets according to a previously described protocol (9). Nucleotide sequences from *P. damselae* subsp. *piscicida* strain 91-197 were determined using the Pacific Biosciences (PacBio) RS II sequencing platform and analyzed by Macrogen Japan (http://www.macrogen-japan.co.jp). The sequence data were assembled using HGAP2, Falcon, and PBcR, and gaps were manually closed by conventional PCR. Genome annotation was conducted using the Prokka Genome Annotation BaseSpase application (10).

The total numbers of reads and bases obtained from strain 91-197 were 165,413 and approximately 1,426 Mb, respectively. The genome of strain 91-197 consists of two circular chromosomes—Ch1 (3,172,118 bp, 41.6% G+C content) and Ch2 (1,054,589 bp, 39.3% G+C content)—and two plasmids—p91-197-1 and p91-197-2—with a total size of 66,468 bp. Ch1 encodes 3,212 coding sequences (CDSs), 129 tRNAs, and 47 rRNAs, and Ch2 encodes 1,496 CDSs and three tRNAs. Plasmids p91-197-1 and p91-197-2 are 37,140 bp and 29,328 bp long and encode 47 CDSs and 30 CDSs, respectively. Ch1 of strain 91-197 is 39,969 bp longer than Ch1 of the Japanese strain OT-51443 (GenBank accession no. BDMQ01000001) and contains 48 more CDSs and three more rRNAs than those of strain OT-51443. Ch2 of strain 91-197 is 43,997 bp longer than that of strain OT-51443 (BDMQ01000002) and is 67 CDSs larger than that of strain OT-51443.

### Accession number(s).

The complete genome sequence of *P. damselae* subsp. *piscicida* strain 91-197 was deposited in DDBJ/GenBank under accession numbers AP018045 to AP018048 (total of four entries for Ch1, Ch2, p91-197-1, and p91-197-2).
